# A Critical Overview of Predictors of Heart Sparing by Deep-Inspiration-Breath-Hold Irradiation in Left-Sided Breast Cancer Patients

**DOI:** 10.3390/cancers14143477

**Published:** 2022-07-18

**Authors:** Gianluca Ferini, Vito Valenti, Anna Viola, Giuseppe Emmanuele Umana, Emanuele Martorana

**Affiliations:** 1 REM Radioterapia srl, Viagrande, I-95029 Catania, Italy; gianluca.ferini@grupposamed.com (G.F.); vito.valenti@grupposamed.com (V.V.); 2 Fondazione Istituto Oncologico del Mediterraneo, Department of Radiation Oncology, Viagrande, I-95029 Catania, Italy; 3 Trauma Center, Gamma Knife Center, Department of Neurosurgery, Cannizzaro Hospital, I-95125 Catania, Italy; umana.nch@gmail.com; 4 IOM Ricerca srl, Viagrande, I-95029 Catania, Italy; emanuele.martorana@grupposamed.com

**Keywords:** deep inspiration breath hold radiotherapy, respiratory gating, breast cancer, adjuvant radiotherapy, anatomical predictors, cardiac risk, radiation-induced CVD risk, adverse radiation effects

## Abstract

**Simple Summary:**

Adjuvant radiotherapy could damage the heart in left-sided breast cancer patients. The deep-inspiration-breath-hold technique may limit the heart exposure to radiation. As non-beneficiaries exist, there is some need to do an upfront cost-effective selection. Some easy-to-use anatomical predictors may help insiders in the treatment decision. The awareness of such findings may improve the efficiency of practitioners’ workflows.

**Abstract:**

Radiotherapy represents an essential part of the therapeutic algorithm for breast cancer patients after conservative surgery. The treatment of left-sided tumors has been associated with a non-negligible risk of developing late-onset cardiovascular disease. The cardiac risk perception has especially increased over the last years due to the prolongation of patients’ survival owing to the advent of new drugs and an ever earlier cancer detection through screening programs. Improvements in radiation delivery techniques could reduce the treatment-related heart toxicity. The deep-inspiration-breath-hold (DIBH) irradiation is one of the most advanced treatment approaches, which requires specific technical equipment and uses inspiration to displace the heart from the tangential radiation fields. However, not all patients benefit from its use. Moreover, DIBH irradiation needs patient compliance and accurate training. Therefore, such a technique may be unjustifiably cumbersome and time-consuming as well as unnecessarily expensive from a mere healthcare cost point of view. Hence the need to early select only the true beneficiaries while tailoring more effective heart-sparing techniques for the others and streamlining the workflow, especially in high-volume radiation oncology departments. In this literature overview, we collected some possible predictors of cardiac dose sparing in DIBH irradiation for left breast treatment in an effort to provide an easy-to-consult summary of simple instruments to insiders for identifying patients actually benefitting from this technique. We critically reviewed the reliability and weaknesses of each retrieved finding, aiming to inspire new insights and discussions on this much-debated topic.

## 1. Introduction

Radiotherapy is an integral part of the therapeutic algorithm for breast cancer (BC) patients following conservative surgery [1] but it is also capable of leaving traces of its administration [2]. The treatment of the left sided tumors (LBC) gives rise to concern regarding the radiation-induced risk of late-onset cardiovascular disease (CVD) according to the Darby’s findings [3], especially considering that breast cancer is often associated with another cardiovascular risk factor such as obesity [4]. Given that new drugs and the cutting-edge knowledge of subcellular mechanisms may help to prolong survival even among patients progressing to metastatic stage, the delayed CVD risk is real in an increasingly large LBC patient population [5,6]. The past decades have led to great improvements in radiation delivery techniques to reduce treatment toxicities in challenging tumor sites close to radiosensitive organs at risk (OARs) [7,8,9,10]. As regards the treatment of LBCs, the most promising and popular technique is represented by the deep-inspiration-breath-hold (DIBH) irradiation, which uses the lung expansion to displace the heart silhouette from the posterior edge of the tangential radiation fields (TRFs) [11] or from the mid-high isodose lines in cases of volumetric modulated arc therapy (VMAT) use [12]. Such a simple trick is able to significantly decrease the heart exposure to radiation in most but not all LBC patients [13]. Since this technique requires specific training and patient compliance, it may be overly cumbersome [14]. So, an upfront selection of those LBC patients actually benefitting from the DIBH technique could be useful to streamline workloads within high-volume departments while discarding the ones without significant variation in heart dose.

This paper aims to offer a literature overview on the predictors of the DIBH-related heart sparing while addressing criticisms and unresolved issues.

## 2. Literature overview

The search strategy for this overview was based on the scanning of works retrieved by PubMed/Medline using “DIBH” and “predict” terms.

### 2.1. Breast Size and Chest Wall Separation

In the work by Xin et al. [15] a comparison between 155 left-sided breast cancer patients treated with adjuvant breath-hold (BH) (82) or free-breath (FB) radiotherapy (73) was made also in order to evaluate whether the dosimetric differences correlate with breast size. As expected, for equivalent planning target volume (PTV) coverage, the maximum (Dmax) and mean heart (Dmean) doses as well as the cardiac volumes receiving low and intermediate doses (from 5 to 30 Gy) were significantly lower in the BH cohort. Interestingly, an increasing target volume worsens the dosimetric parameters of the heart and left lung in both groups. As this finding was significant only in the BH group, the authors concluded that the patients benefitting more from BH irradiation technique are the ones with small left breast size. The same authors reported that an increasing left lung volume decreased significantly the radiation exposure of the heart only in the BH group. However, they did not search for any correlation between the target size, lung expansion and dosimetric parameters of heart. Indeed, the investigation was conducted only as an inter-patient comparison, while omitting any intra-patient assessment of variations in the dosimetric parameters of the heart and lung between the BH and FB settings. A greater lung capacity involves a more effective displacement of the heart from the radiotherapy target. Therefore, the finding regarding the inverse correlation between the breast volume and the reduction of heart exposure to radiation in BH setting risks being imprecise or even misleading if not associated with a concomitant evaluation of respiratory motion of the left breast. In fact, from another point of view, patients with larger left breast could be the ones who deserve a specific respiratory training to reduce cardiac doses as much as possible. Probably, the largest breast volumes determined a longer and deeper CWS that enclosed a greater heart volume within the posterior edge of the two opposing TRFs compared to the smallest volumes. After all, the lack of an intra-patient comparison did not allow to appreciate the real gain related to the BH irradiation with respect to a FB one among patients with larger target volume. To weaken further the findings of this experience, there is a limited variety in body mass index (BMI) and breast size.

Ferini et al. [16] conducted an intra-patient assessment between FB and DIBH dosimetric data in a cohort of 116 LBC cases. They found that an adequate dose coverage of larger PTVs generally need longer distance between the medial and lateral entry points of the TRFs (namely the chest wall separation, CWS, in other reports), which consequently cross a greater heart volume, as in cases of pendulous breasts. For these reasons, patients that benefitted most of DIBH were the ones with large PTVs, especially those with volumes >647 cc and CWS >22.4 cm.

### 2.2. The Cardiac Contact Distance in the Axial (CCDax) and Parasagittal (CCDps) Planes and the Lateral Heart-to-Chest Distance (HCD)

Cao et al. [17] found some thoracic anatomical parameters to correlate with the measured heart dose reduction under the DIBH conditions. In particular, the authors examined three distances on the free-breath computed tomography (CT) simulation in 67 left-sided breast cancer patients: the cardiac contact distance in the axial plan (CCDax), the cardiac contact distance in a parasagittal plan (CCDps), and the lateral heart-to-chest distance (HCD). For the latter two, a positive and a negative linear correlations, respectively, with the DIBH-related Dmean_heart reduction were proven and cross-validated: the benefit from DIBH increases with increasing CCDps and with decreasing HCD. CCDps and HCD were not interdependent and could be used to predict the heart dose sparing in DIBH by employing their ratio (FB-CCDps/FB-HCD). Interestingly, the CCDax did not correlate with Dmean_heart reduction in DIBH. This might mean that the CCDax is less sensitive than CCDps to the lung volume change following breath hold. Such an assumption can be important at least for two reasons: (1) CCDax is not to be considered as a surrogate of CCDps; (2) the lack of a proportional relationship between the CCDax and the heart dose reduction in DIBH could be due to the fact that the axial slice where the CCDax is measured may not be representative of the magnitude of the heart displacement from the chest wall with DIBH, in a way that the parasagittal view predicts the DIBH-related cardiac sparing much better than the axial one. The latter consideration spawns the next: some sections of the left anterior descending artery (LAD) could not be far enough away from the chest wall within the treatment fields in DIBH so to still receive a significantly harmful dose. Indeed, these authors focused exclusively on the Dmean_heart reduction achieved by DIBH while omitting any information about the DIBH effect on the LAD maximum dose (Dmax_LAD), which is the type of constraint to evaluate in serial OARs [18]. In fact, a focal damage in this vital anatomical structure may obviously have hazardous consequences [19]. So, even if the simple method proposed by these authors is undoubtedly useful for the cardiac risk assessment, this could be incomplete in the absence of data about the LAD dose.

Rochet et al. [20] evaluated the FB-CCDax and FB-CCDps as predictors of cardiac radiation exposure among a small cohort of LBC patients (35), of whom only 75% reported a dosimetric benefit from DIBH, meaning a Dmean_heart reduction ≥ 0.9 Gy. These authors found no correlation between FB-CCDax and any cardiac dosimetric indexes. On the other side, FB-CCDps was significantly associated with radiation doses to the heart and its substructures (left ventricle and LAD): the longer the FB-CCDps, the higher the dose. Nonetheless, this parameter was not able to predict the dose reduction achieved with DIBH over FB. Indeed, a constant ∆ of 16 Gy in heart equivalent uniform dose was reported for the entire range of FB-CCDps. Although the DIBH-induced advantage was higher for FB-CCDps > 2 cm, there were some cases of significant dose reduction even for values <2 cm. Therefore, the authors suggested this parameter as a tool to identify patients with unfavorable cardiac anatomy without further qualifying an optimal cutoff value to filter out cases not benefiting from DIBH planning. The latter purpose was likely impeded by the small sample size.

### 2.3. Heart Volume in Field

Register et al. [21] found the change in Heart Volume In Field (∆HVIF) as the only independent predictor of cardiac sparing, although almost all measured anatomical parameters significantly correlated with most heart and LAD dosimetric parameters in a small cohort of LBC 64 patients. ∆HVIF is of course inferable only after performing both FB and DIBH CT scans and its assessment needs at least a basic arrangement of TRFs, albeit without plan optimization. Interestingly, these authors were able to identify a subgroup of 19 patients minimally benefitting from DIBH. Some of these patients (11/19) shared an HVIF ≤ 1 cc on FB indicating a favorable baseline anatomy, which can be summarized as follows: low BMI, short chest separation (CS), and length of contact between the heart and chest wall (HCWL). Other three patients had suboptimal breath-hold while the remaining five simply had no meaningful anatomic changes despite DIBH.

The above findings are concordant with those reported by Ferdinand et al. [22] In this even smaller series of LBC patients (31), the reduction in DIBH Dmean_heart could be predicted by the ∆HVIF. However, no parameter was able to predict a reduction in Dmax_LAD. These authors suggested a ∆HVIF threshold of 6 cc to predict a DIBH reduction in Dmean_heart of at least 20%. In this report, despite not being a significant predictor, a ∆-maximum heart depth (∆MHD) of 7 mm implied a reduction of at least 20% in DIBH Dmean_heart, reaching 50% for values greater than 1 cm.

Moreover, in the report from Kim et al. [23], the ∆HVIF could predict the mean heart and LAD dose reductions with DIBH, reaching 44% and 67%, respectively, as compared to FB. These authors found a linear correlation between the ∆HVIF and mean dose reductions that can be summarized in two formulas: dose reduction (cGy) = 8.1 (cGy/cc) × ∆HVIF (cc) + 26.0 (cGy) for Dmean_heart and dose reduction (cGy) = 81.6 (cGy/cc) × ∆HVIF (cc) + 109.1 (cGy) for mean LAD dose (Dmean_LAD). Even the *z*-axis sternal displacement correlated with mean dose reductions, just like the ∆HVIF. By using this parameter, the two formulas for predicting the mean dose reductions became: dose reduction (cGy) = 1.4 (cGy/mm) × sternal displacement (mm) + 35.6 (cGy) for Dmean_heart and dose reduction (cGy) = 21.2 (cGy/mm) × sternal displacement (mm) + 102.4 (cGy) for Dmean_LAD. The measurement of sternal displacement by a simple clinical evaluation does not require performing two CT scans (FB and DIBH), contrary to the ∆HVIF, and therefore allows to select in advance patients with low values (i.e., <1 cm, for who not significant dose reductions are expected from DIBH use) for alternative treatments, such as prone irradiation or intensity modulated radiation therapy (IMRT). Conversely, a measured excursion >1 cm could avoid the need for a FB-CT scan since the DIBH-related dosimetric advantage should be significant. However, caution is required when adopting this empirical method that could be subject to potentially misleading approximations.

### 2.4. BMI, the Seasonal Effect, and Lung Predictors

In the work of Tanguturi et al. [24], younger age, higher BMI, and larger ∆LungVol (that is the change in lung volume between FB and DIBH) could act as independent predictors for ∆-Dmean_heart between DIBH and FB plans. The authors assumed that age and BMI could affect the dynamic interactions of thoracic organs during the respiratory cycle. No correlation between BMI and clinical target volume (CTV) size was searched for. Thirty-eight patients were treated in FB and 110 in DIBH. The first were on average older and sicker (lung and heart diseases) than the second. It is worthwhile to note that 39 DIBH plans lead to neutral changes (25) or to >20 cGy increase (14) in mean heart dose with respect to the paired FB plans. Apart from the general indications about age, BMI, and ∆LungVol, no specific characterization of the non-benefitting subgroup was made.

Mkanna et al. [25] proved that higher BMI and spring/winter timing of CT simulation were associated with larger mean heart dose and V4 (volume of heart receiving 4 Gy) differences between FB and DIBH plans. Since almost the entire cohort of 103 patients underwent mastectomy (97%), the effect of BMI on the heart sparing by DIBH was independent of breast size. This supports the idea that the body habitus in itself conditions the spatial relationship between the heart and chest wall. As regards the seasonal effect, this could reflect the decline in lung function during summer/fall. The authors acknowledged the lack of dosimetric analysis of sub-cardiac structures (i.e., LAD) as a study limitation.

Koide et al. [26] analyzed various spirometry parameters in 100 LBC patients and found only the vital capacity (VC) as a significant preoperative predictor of Dmean_heart reduction using DIBH. This parameter agreed with the predictive ability of the lung volume on FB-CT scan (LV-FB), thus eliminating the need for radiation exposure to appropriately select DIBH-beneficiaries. Interestingly, VC lost accuracy at the lowest values and with increasing age. This could be due to the impossibility for spirometry to measure the residual volume (RV), so that the VC may not be representative of LV-FB in cases of large RV, as commonly occurs in older people. In these cases, the VC-based prediction may slightly overestimate the heart sparing due to a lower lung expansion and cardiac displacement from the chest wall during DIBH than expected. Conversely, BMI did not correlate with mean heart dose and was unable to predict its reduction through DIBH.

The above findings agree with those reported by Dell’Oro et al. [27] that showed as the total lung volume measured on a FB plan could potentially be utilized to predict cardiac exposure and assist with patient selection for DIBH.

### 2.5. Maximum Heart Depth and Tumor Bed Site

Among 134 LBC patients examined by Tanna et al. [28], only 28 would have plausibly benefitted from DIBH as a result of having a Dmean_heart > 3 Gy in FB. These authors tested two different methods for an upfront selection of DIBH-beneficiaries in an effort to save time by eliminating the need for performing two CT scans (DIBH and FB) and planning both of them. The first method was based on candidating the patients with ≥1 cm FB-MHD to DIBH. The second method involved patients with a tumor bed in the inferior portion of the breast or whose tumor bed extends over two quadrants or those undergoing chest wall radiotherapy or, if none of these, with ≥1 cm FB-MHD to be candidates to upfront DIBH. Both methods selected 66 patients, thus overestimating the FB-Dmean_heart in 40 and 42 cases, respectively, and failed to detect 2 and 4 patients with >3 Gy FB-Dmean_heart, respectively. These findings mean that both methods have a good sensitivity (≥86%), poor specificity (≈60%), and a low positive predictive value (<40%). Additionally, the second method allows to avoid conducting two CT scans in the majority of patients with a selection performance similar to that of the first method and to appropriately exclude a large proportion of patients not needing DIBH. However, the safety threshold of 3 Gy Dmean_heart was totally arbitrary, as recognized by the authors themselves. Interestingly, by adopting this criterion, only a small proportion (28/134) of LBC patients would have benefitted from DIBH.

**Figure 1:**
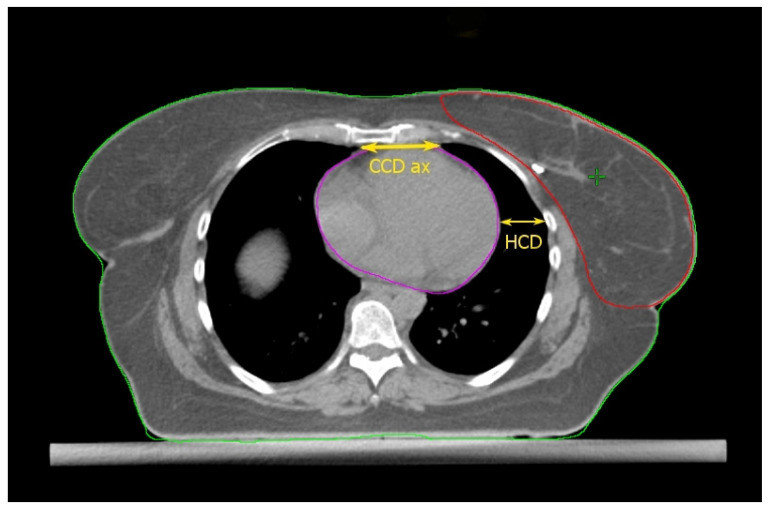
Anatomical parameters on the axial CT plane passing through the liver dome. Read the text for the explanation.

**Figure 2:**
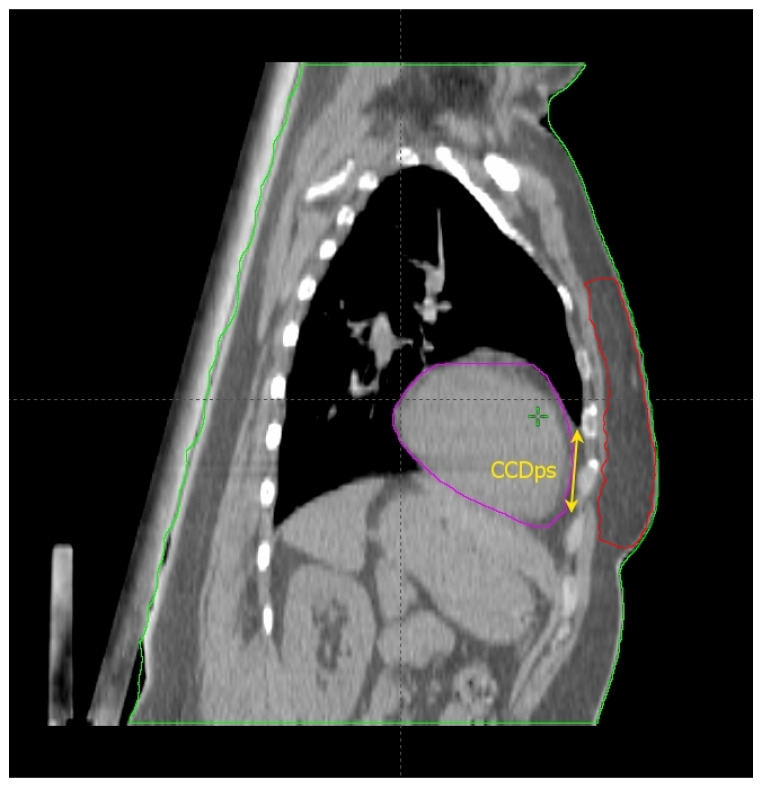
Anatomical parameters on the parasagittal CT plane, chosen as described in the text.

**Figure 3:**
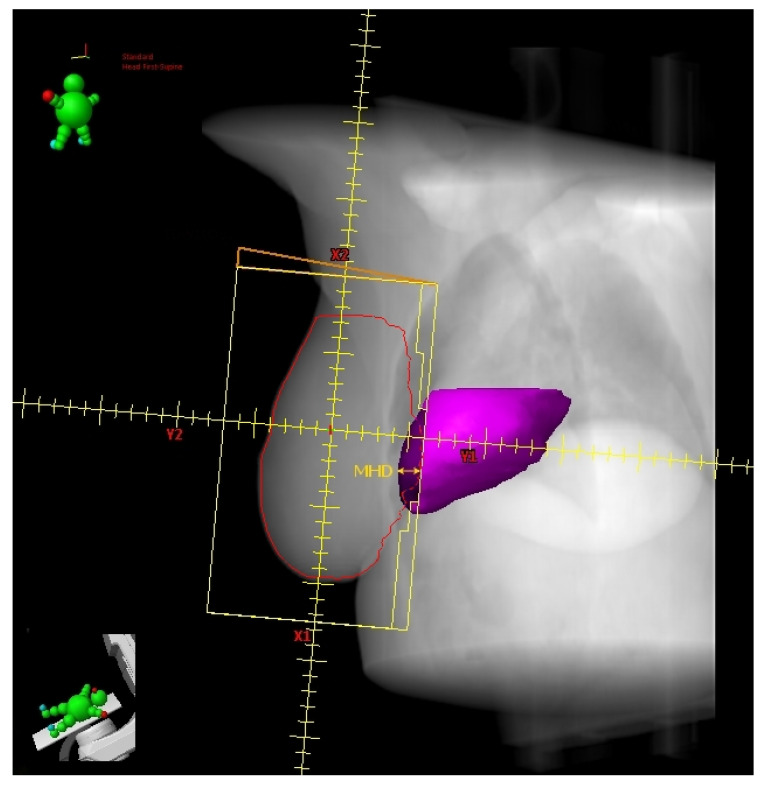
Digitally reconstructed radiograph (DRR) with external tangential beam’s-eye-view. The heart volume in field (HVIF, dark magenta) and the maximum heart depth (MHD) have been highlighted.

**Figure 4:**
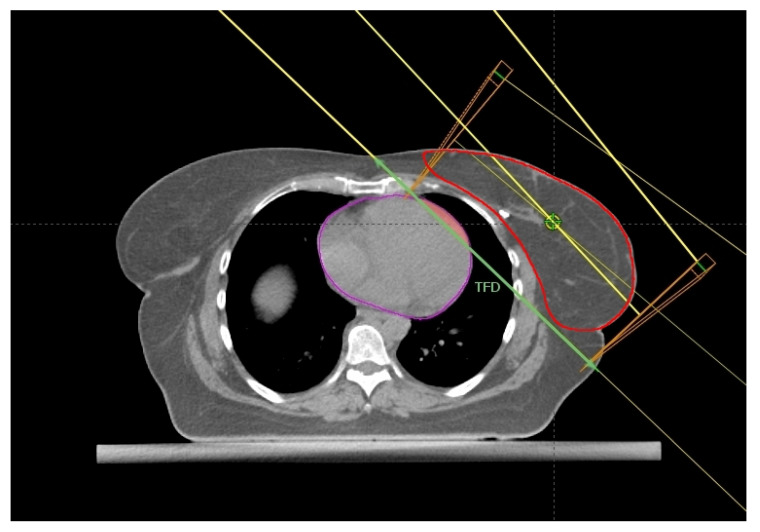
Axial CT plane passing through the liver dome with a standard tangential beam arrangement. The tangential field distance (TFD, green line), HVIF (pink area), and the CTV (red) have been highlighted.

The above studies are collected in Table 1; the anatomical predictors are shown in Figure 1, Figure 2, Figure 3 and Figure 4.

## 3. Further Considerations

The goal of heart sparing by DIBH is especially mandatory in the presence of one or more baseline cardiac risk factors that further worsen the CVD risk deriving from the FB radiation dose delivery. As regards the latter prediction, the DIBH planning would seem to ensure a relative risk decrease up to 64.7% [29] by taking as reference the work by Darby et al. [3].

According to the computing model elaborated by Corradini et al. [12], the estimated CVD risk was significantly lower for the DIBH 3D-CRT plan than all other CT-simulation conditions (FB 3D-CRT, DIBH VMAT, FB VMAT plans). The risk reduction from DIBH was more prominent among older patients (>70 years) due to a higher frequency of worse baseline cardiac risk factors. Interestingly, no significant difference in estimated CVD risk between FB plans (VMAT vs. 3D-CRT) was documented. Moreover, DIBH 3D-CRT was significantly better than DIBH VMAT, so calling into question the safety issue of the low-dose bath deriving from the arrangement of VMAT arcs.

DIBH is not always the best technical trick to reduce dose to the heart. Indeed, the left breast irradiation in the prone position might be more effective than supine DIBH for heart sparing. For example, in the work by Wang et al. the first radiation delivery modality was more advantageous than the second in 62.1% of cases [30]. The dosimetric gain was more pronounced for highly pendulous and large breasts. Again, these findings focus attention on breast area rather than non-breast-related patient characteristics such as thorax shape, through which it is not possible to predict the magnitude of the DIBH-related gain [31]. To distinguish which patients benefit most of prone positioning, some authors developed a prediction algorithm based on anatomical features extracted from supine FB-CT scan with the aim of early selecting the best treatment position [32]. Moreover, proton therapy may be more cost-effective than DIBH-RT in selected LBC cases [33].

As demonstrated by Ward et al. [34] by adequately selecting gantry angles and introducing a dosimetric goal of Dmean_heart < 4 Gy in the treatment plan optimization of a hybrid IMRT technique using tangential/non-tangential fields, it is possible to drastically reduce the FB-heart radiation exposure without compromising overall dose coverage of breast PTV even in the LBC patients, so much so to avoid the need for DIBH.

The reproducibility of DIBH amplitude is essential for the heart dose distribution to not deviate from the planned one, as even minimal shifts may determine significantly detrimental changes in Dmean_heart [35].

The close proximity between the target and OAR would require a stereotactic-like radiation delivery to achieve an effective heart sparing. This is hampered by the fact that both heart due to its own beat and chest wall may slightly move during DIBH [36]. Therefore, the planned dose could not fully match the actually delivered. Indeed, some respiratory gating systems, such as the Varian RPM™ one, work normally with the beam-on even after reaching the DIBH peak when a minimal expiratory flow may occur, corresponding to a vertical displacement of the chest surface up to 0.6–1 cm with unforeseeable repercussions on heart and LAD doses. That is why the most advanced systems based on the surface guidance should be more robust [37]. Moreover, when the inter-fraction setup verification is based on matching the breast silhouette between the digitally reconstructed radiographs (DRRs) and the electronic portal images, it can be misled by the development of deforming breast edema during radiotherapy, resulting also in an altered radiation exposure of the heart and its substructures (LAD) with respect to the estimated one in treatment planning. The LAD is not always easily detectable as well as the anterior interventricular groove where it stands. As a result of this, the LAD exposure to radiation could be over- or under-estimated.

The Dmean_heart is one of the most common parameters evaluated by radiation oncologists during plan optimization, but it may not reflect any high doses in specific heart subvolumes (i.e., left ventricle and its segments), which could carry a great CVD risk even in the presence of acceptable Dmean_heart [38,39,40].

Lastly, new knowledge-based treatment planning softwares based on machine learning algorithms, if adequately trained with large and varied datasets, could greatly simplify the selection process of patients actually needing for DIBH with significant time savings. With this strategy, Rice et al. were able to accurately predict the Dmean_heart on which to customize the treatment choice [41].

The prediction model drawn up by Xu et al. [42] was developed from a kernel density estimation of dose-volume histograms (DVHs) of FB- and DIBH-IMRT plans by extracting features from a reference training dataset according to a knowledge-based approach (machine learning-like). The DVH prediction worked only within the same breath setting: the FB and DIBH models were able to predict the FB and DIBH clinical plan dosimetries, respectively, but each model was unable to predict the reverse respiratory condition (FB → DIBH and vice versa), hence the need for two CT scans in any case. By contrast, Koide et al. [43] synthesized a DIBH-CT model starting from the corresponding FB-CT planning with a good consistency between the predicted mean heart dose and the actually planned. This might allow to candidate to the additional DIBH-CT scan only the cases who actually benefit from it, with consequent saving of resources and time.

Currently, the heart sparing deriving from DIBH irradiation may be regarded as an axiom without clinical validation. Indeed, no study clinically confirmed the DIBH benefit in terms of reduction of CVD risk. Conversely, some reports failed to detect any significant difference in cardiac muscle perfusion imaging (SPECT) among LBC patients submitted to RT with or without DIBH: both groups showed reduction of apical cardiac perfusion. This finding raises doubts about the harmlessness of the lower radiation doses carried by the DIBH technique [44].

None of the findings here discussed prevails over the others because their reliability should be tested and validated in much larger cohorts. However, this paper offers a comprehensive and useful overview of predictors to be implemented in routine clinical practice to better organize the therapeutic proposal for LBC patients needing adjuvant RT. Moreover, updated consensuses on the best management of cardiac issues among cancer patients can properly assist the breast radiation oncologist’s work [45].

## 4. Conclusions

Not all LBC patients benefit equally from DIBH technique. An upfront selection of the main beneficiaries by using simple predictors could allow to well-organize the workload of high-volume departments. Large external datasets are needed to cross-validate the findings here summarized. The dosimetric improvements should also be clinically confirmed to assess the appropriateness and reliability of this irradiation technique and to state how many treatments are necessary to prevent a radiation-induced cardiac death within healthcare cost-effectiveness analyses.

## Figures and Tables

**Table 1:** The most relevant findings of the above cited studies.

Authors	N° of LBC Patients	Irradiation Technique	Highlights
Xin et al. [15]	155 (82 DIBH; 73 FB)	3Dimensional-Conformal Radiation Therapy (3D-CRT, tangential fields)	Dmax, Dmean, and cardiac volumes receiving from 5 to 30 Gy are lower in the DIBH cohort compared to FB one. Patients with smaller left breast size benefit more from DIBH irradiation. (misinterpretation of the finding?) An increasing left lung volume decreased significantly the radiation exposure of the heart only in the BH group.
Ferini et al. [16]	116	3D-CRT (tangential fields)	Significant isodose lines cross a greater heart volume with increasing PTV as this requires a greater distance between medial and lateral entry points of TRF.
Cao et al. [17]	67	3D-CRT (tangential fields)	The benefit from DIBH increases with increasing CCDps and decreasing HCD measured on the free breath CT simulation. CCDax did not correlate with the Dmean_heart reduction in DIBH.
Rochet et al. [20]	35	3D-CRT (tangential fields)	No correlation between FB-CCDax and dosimetric indices. A longer distance of FB-CCDps is associated with an higher radiation dose.
Register et al. [21]	64	3D-CRT (tangential fields)	ΔHVIF is an independent predictor of cardiac sparing. A free breath HVIF ≤ 1cc suggests a minimal benefit from DIBH.
Ferdinand et al. [22]	31	3D-CRT (tangential fields)	Above a ΔHVIF threshold of 6cc it can be assumed a reduction in Dmean_heart by DIBH of at least 20%. Above a ΔHVIF threshold of 13cc it can be assumed a reduction in Dmean_heart by DIBH of at least 50%.
Kim et al. [23]	97	3D-CRT (tangential fields)	Empirical method that uses the ΔHVIF and/or inspiratory sternal displacement to predict the DIBH mean dose reductions in the heart and LAD.
Tanguturi et al. [24]	148 (110 DIBH; 38 FB)	3D-CRT (tangential fields)	Age and BMI are involved in dynamic interactions of thoracic organs during respiratory cycle.
Mkanna et al. [25]	103	3D-CRT (tangential fields)	Higher BMI and spring/winter treatment (seasonal effect in lung function) were associated with larger FB/DIBH differences in mean heart dose and volume of heart receiving 4 Gy.
Koide et al. [26]	100	3D-CRT (tangential fields)	Vital capacity (a spirometric parameter) is a significant predictor of Dmean_heart reduction using DIBH but loses accuracy at lower values or with increasing age.
Dell’Oro et al. [27]	20	3D-CRT (tangential fields)	Total lung volume predicts cardiac exposure and is useful in patient selection for DIBH.
Tanna et al. [28]	134	Forward planned Intensity Modulated Radiation Therapy (IMRT)	Patients with maximum heart depth ≥ 1 cm or those with any of the following: ⚬ Tumor bed in the inferior portion of the breast; ⚬ Tumor bed extends over two quadrants; ⚬ Undergoing chest wall radiotherapy; are candidates for DIBH.
